# Enzymatic Nanomotors Integrated with Plant Extracts: Biochemical Mechanisms, Applications, and Clinical Perspectives

**DOI:** 10.3390/molecules31132344

**Published:** 2026-07-03

**Authors:** Joanna Lemanowicz, Kinga Gawlińska, Iwona Jaskulska, Emilia Leśniak, Antoni Kuczyński

**Affiliations:** 1 Division of Biochemistry, Faculty of Medicine, Bydgoszcz University of Science and Technology, Bernardyńska 6 St., 85-029 Bydgoszcz, Poland; emiles001@pbs.edu.pl (E.L.); antkuc000@pbs.edu.pl (A.K.); 2 Department of Clinical Pharmacy, Medical College, Jagiellonian University, Medyczna 9, 30-688 Krakow, Poland; kinga.gawlinska@uj.edu.pl; 3 Department of Agronomy and Food Processing, Faculty of Agriculture and Biotechnology, Bydgoszcz University of Science and Technology in Bydgoszcz, 7 Kaliskiego St., 85-796 Bydgoszcz, Poland; iwona.jaskulska@pbs.edu.pl

**Keywords:** enzymes, nanomedicine, plant extract, therapeutic and catalytic nanomotors, targeted drug delivery, precision medicine

## Abstract

Enzymatic nanomotors (EMNMs) represent an emerging class of intelligent nanosystems that exploit enzymatic biocatalysis to generate autonomous motion within biological environments, including complex cellular and tissue contexts within living organisms. Owing to their ability to utilize endogenous biofuels, high biocompatibility, and capacity for targeted propulsion, EMNMs have demonstrated considerable potential in diverse biomedical applications. These include targeted drug delivery, cancer therapy, diagnostics, and bioimaging, as well as the traversal of biological barriers. This review comprehensively discusses the mechanisms underlying enzyme-driven propulsion, nanomotor design strategies, and their current and prospective applications in medicine, while also addressing major challenges associated with enzymatic stability, biocompatibility, motion control, and clinical safety. Furthermore, future perspectives are highlighted, including enzyme cascade systems, intelligent nanomotor swarms, biodegradable materials, and strategies facilitating clinical translation. As a representative example of practical application, curcumin was employed as a model therapeutic agent due to its well-established anticancer, anti-inflammatory, and antioxidant properties, enabling evaluation of the nanomotors’ capability for controlled, pH-responsive release of therapeutic cargo. Nanophytomedicine enhances the therapeutic efficacy of phytochemicals by improving their stability, bioavailability, and targeted delivery through nanocarrier systems. The integration of phytotherapy with nanotechnology offers promising opportunities for the development of safer and more effective therapeutic strategies.

## 1. Introduction

Nanotechnology focuses on the properties and modification of materials at the nanoscale, enabling the precise manipulation of material structures. This has facilitated the development of advanced solutions that were previously unattainable at the macroscale, including innovative drug delivery systems and targeted therapies [1]. In nanomedicine, increasing attention is being directed toward the development of active transport systems capable of functioning in complex biological environments, improving biofilm penetration, enhancing diagnostic accuracy, and increasing therapeutic efficacy while minimizing treatment-related side effects [2,3].

Cells play a crucial role in this context, as they constitute natural barriers that nanomaterials must overcome. At the same time, they serve as models for the design of biomimetic nanocarriers that exploit cellular transport mechanisms for precise drug delivery. The application of biomimetic strategies based on cell membranes has been shown to significantly improve the ability of nanocarriers to penetrate biological barriers and prolong their circulation time in vivo. Moreover, this approach enables the targeted delivery of therapeutic payloads, as demonstrated in recent studies on nanocarriers for nanomedicine applications [4].

Nanotechnology focuses on the properties and modification of materials at the nanoscale, enabling the precise manipulation of material structures. This has facilitated the development of advanced solutions that were previously unattainable at the macroscale. Owing to their unique size, structure, physicochemical properties, and ability to achieve autonomous motion through diverse propulsion mechanisms, micro/nanomotors (MNMs) have attracted significant attention across various scientific disciplines. Their potential applications span medicine, environmental remediation, propulsion and energy systems, as well as the development of hybrid structures [5,6,7,8,9,10].

The origins of micro- and nanomotors can be traced back to pioneering studies published by researchers from Pennsylvania State University [11] and University of Toronto [12]. These studies represent important milestones in the development of synthetic nanomotors, demonstrating both linear motions driven by chemical propulsion or oxygen gradients and the rotational behavior of nanoparticles. In both cases, hydrogen peroxide decomposition served as the chemical “fuel” powering motor activity. Over the past two decades, continuous advances in research and technology have enabled the fabrication of increasingly sophisticated and intelligent nanomotors from a wide range of materials. In parallel, the development of advanced computational methods and artificial intelligence (AI) tools has provided new opportunities for optimizing nanomotor design and addressing complex biomedical and engineering challenges [13,14].

Nanomedicine is an interdisciplinary field that applies nanomaterials in diagnostics, therapy, drug delivery systems, and tissue engineering and regeneration [15]. This area of research is developing rapidly and is increasingly being applied in the treatment of cancer, infectious diseases, and cardiovascular disorders. Moreover, a growing number of therapeutics are currently being developed in nanoscale formulations [16]. Despite these advances, the successful translation of nanomedicine-based technologies into clinical practice requires strict compliance with rigorous standards related to safety, quality, and manufacturing process control [17].

**Figure 1:**
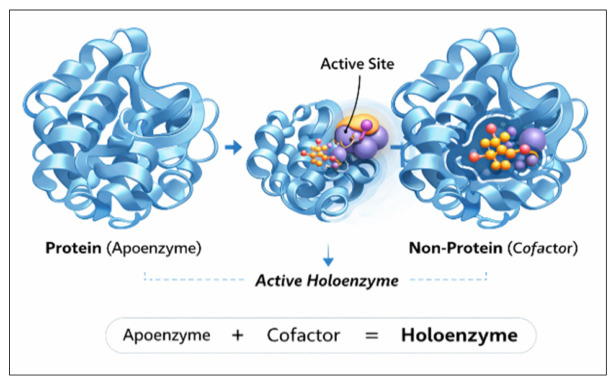
Formation of an active holoenzyme through cofactor binding. The binding of a non-protein cofactor to the inactive apoenzyme generates an active holoenzyme by completing the enzyme’s active site and enabling catalytic activity.

Enzymes are biological catalysts. They accelerate chemical reactions by lowering the activation energy barrier without being consumed in the process [18,19,20]. They are environmentally and biologically compatible and can serve as natural alternatives to chemical catalysts [21]. The mechanism of enzyme action is based on the specific binding of the substrate to the enzyme’s active site. This allows for its conversion into a product and the subsequent release of the enzyme, which can then catalyze additional reactions. This catalytic process facilitates the conversion of chemical energy into mechanical energy, thereby supporting the self-propulsion mechanisms observed in enzyme systems [22]. Most enzymes are proteins with a tertiary or quaternary structure. This enables specific substrate recognition due to the unique geometry and physicochemical properties of their active sites. In some cases, enzymatic activity requires the presence of cofactors that enhance catalytic efficiency (Figure 1) [23,24]. Enzymes participate in nearly all metabolic processes in living organisms, including digestion, DNA synthesis, and the regulation of biochemical pathways. There is also growing interest in “nanozymes”-nanomaterials that mimic enzyme-like catalytic activity-which may further expand the applications of biocatalysis beyond thosef of natural enzymes [25].

Thanks to the ability of enzymes to self-propel, the concept of so-called enzymatic nanomotors (EMNM) is emerging [22]. Combining enzymes with nanomotors can increase enzyme stability and protect them from degradation. This enables their reuse and precise transport to specific locations. As a result, the potential applications of biocatalysis are significantly expanded. Enzymatic motors show promise for in vivo applications due to their biocompatibility. Early studies have shown that enzymes such as catalase (CAT), urease, lipase, and glucose oxidase (GOx) can generate propulsive forces, causing nanoparticles to move in the presence of a substrate [2]. These mechanisms involve both the generation of reaction product concentration gradients and local changes in pressure or enzyme conformation [26]. Advances in this area open up new possibilities for medical applications, including targeted drug delivery, cancer therapy, imaging and diagnostics, and overcoming biological barriers.

The integration of plant-derived compounds, such as curcumin, resveratrol, quercetin, catechins/EGCG, berberine, and paclitaxel, together with enzymes as bioactive components of nanomaterials and nanomotors, has emerged as a promising strategy for improving their stability, bioavailability, and targeted delivery. Such hybrid systems enable controlled drug release and site-specific therapeutic activity by combining the pharmacological potential of phytochemicals with enzyme-driven active transport mechanisms. Consequently, these approaches highlight new directions in the development of advanced, nature-inspired therapeutic platforms and nanomedicine strategies [27].

Many medicinal plants and their extracts exhibit synergistic antioxidant and antimicrobial activities, which, when combined with nanocarrier systems, create new opportunities for advanced therapeutic applications [28]. The crucial role of phytochemical metabolites in the green synthesis of nanoparticles, particularly those based on transition metals, has been widely demonstrated. Secondary metabolites, including polyphenols, terpenes, alkaloids, and saponins, function as biocompatible reducing and stabilizing agents, while also imparting specific biological properties to the resulting nanostructures [29]. These naturally derived compounds represent a more sustainable and environmentally friendly alternative to conventional chemical reagents. Traditional chemical reagents used for metal salt reduction and the prevention of colloidal agglomeration are often toxic and incompatible with the principles of green chemistry [30]. Polyphenols, one of the most abundant groups of natural antioxidants, are of particular importance in the green synthesis of nanoparticles. Owing to the presence of multiple hydroxyl (-OH) groups, they exhibit strong metal ion-reducing capabilities and good solubility in aqueous media. During the reaction process, hydroxyl groups are oxidized to carbonyl (C=O) groups, which contributes to the electrostatic stabilization of the newly formed nanoparticles [31]. In this mechanism, phytochemicals function both as reducing agents and capping agents, enabling control over the morphology and stability of the synthesized nanostructures. Such engineered systems may serve as promising platforms for the controlled delivery of bioactive molecules, including agents used in targeted cancer therapy [32].

The synergistic integration of EMNMs with bioactive compounds derived from medicinal plant extracts enables the development of biohybrid therapeutic systems with enhanced biological activity. These systems combine enzymatic catalysis with phytochemical functions, leading to improved regulation of chemical gradients, oxidative stress, and the bioavailability of active compounds.

This review aims to critically analyze and systematically summarize the mechanisms of action of biohybrid systems based on EMNMs and plant-derived extracts, with particular emphasis on their synergistic interactions, therapeutic potential, and prospects for clinical translation.

## 2. Mechanisms of Enzymatic Propulsion

This section explains the basic enzymes and motor proteins used as nanomotors. It also explains the fundamental physicochemical phenomena that convert biocatalytic energy into mechanical work in EMNMs. Understanding these driving mechanisms is crucial for designing next-generation EMNMs with optimized speeds, trajectory control, and targeted release kinetics for biomedical applications.

### 2.1. Key Enzymes Applied in Enzymatic Nanomotors (EMNMs)

Enzymes such as catalase, urease, glucose oxidase, lipase, as well as other hydrolases and oxidases, have been extensively applied as biological nanomotors to drive the propulsion of micro- and nanoscale particles [2,33,34,35].

**Catalase (CAT)** is a natural oxidoreductase enzyme that catalyzes the decomposition of hydrogen peroxide (H_2_O_2_) into water (H_2_O) and molecular oxygen (O_2_) [36]:

$$2H_{2}O_{2}\to 2H_{2}O+O_{2}$$

Owing to its strong antioxidant properties, CAT plays an important role in protecting cells against oxidative stress [37]. In the field of micro- and nanomotors, CAT is one of the most widely used propulsion enzymes [35]. In CATe-powered systems, the enzymatic reaction generates oxygen, leading to the formation of gas bubbles on the surface of the carrier, which provides the driving force for particle propulsion. The generated oxygen bubbles propel the particles through a bubble-driven mechanism. However, the use of H_2_O_2_ as a fuel is limited under physiological conditions due to its cytotoxicity, which restricts in vivo applications [38]. Despite this limitation, CAT remains one of the most frequently employed enzymes in nanomotor systems. This is due to its exceptionally high catalytic efficiency, which enables intensive oxygen generation even at relatively low H_2_O_2_ concentrations. In addition, its favorable biocompatibility makes CAT particularly attractive for biomedical and biological applications [2].

**Peroxidases** belong to the oxidoreductase class of enzymes and have attracted considerable interest as catalytic components of EMNMs. These enzymes utilize the energy released during the oxidation of various substrates, such as phenolic compounds and hydrogen peroxide (H_2_O_2_), to generate local chemical gradients and asymmetrical distributions of reaction products around the nanoparticle surface. The resulting physicochemical imbalance induces autonomous particle movement through a self-propulsion mechanism [39]. The general reaction catalyzed by peroxidases can be expressed as follows:

$${RH}_{2}+H_{2}O_{2}\to R+2H_{2}O$$

where RH_2_ and R denote the substrate in its reduced form and the oxidized reaction product, respectively. In the presence of an electron donor, such as phenol or NADH, peroxidase-catalyzed reactions involve local substrate consumption and the generation of products exhibiting different diffusion rates. In nanobiotechnological systems, immobilized horseradish peroxidase and related enzymes can promote the movement of nanocarriers along substrate gradients through asymmetric catalytic activity on the particle surface. This uneven distribution of the reaction generates a chemotactic response that drives directional motion of the nanostructures. This propulsion mechanism is based on the concept of “enzymatic diffusion-driven motion,” in which the energy released during a chemical reaction is converted into the translational movement of the nanostructure [40,41].

**Urease,** a member of the hydrolase enzyme class, is among the most extensively studied biocatalysts employed in the design of biologically powered micro- and nanomotors. Urease catalyzes the hydrolysis of urea into ammonia and carbon dioxide. This reaction generates localized chemical gradients that can effectively drive the autonomous motion of nanostructures through a self-diffusiophoretic mechanism [42,43].

$$H_{2}N-CO-NH_{2}+H_{2}O\to 2NH_{3}+CO_{2}$$

The endogenous occurrence of urea in physiological environments makes urease particularly attractive for potential clinical applications. Urease-powered nanomotors exhibit high biocompatibility and considerable stability under biological conditions, which significantly enhances their suitability for biomedical use. Consequently, these systems have demonstrated substantial potential in applications such as targeted drug delivery, immunotherapy, and diagnostic technologies [44,45].

**Glucose oxidase (GOx)** is an oxidoreductase enzyme that catalyzes the oxidation of *β*-D-glucose to D-gluconolactone, accompanied by the simultaneous reduction of molecular oxygen to hydrogen peroxide (H_2_O_2_).

$$C_{6}H_{12}O_{6}+O_{2}\to C_{6}H_{10}O_{6}+H_{2}O_{2}$$

The H_2_O_2_ generated during this reaction can directly contribute to the propulsion of nanostructures or be further utilized by CAT to produce oxygen bubbles that enhance particle motility [46]. Furthermore, GOx based nanomotors have been investigated in combination with natural antioxidants and plant-derived bioactive compounds to improve catalytic stability, biocompatibility, and potential biomedical applicability, particularly in drug delivery and biosensing systems [2,26]. GOx is commonly incorporated into enzymatic cascade systems, particularly for applications within the tumor microenvironment, where elevated glucose concentrations provide an abundant endogenous substrate. Nevertheless, compared with CAT or urease, GOx is less frequently employed as a primary propulsion enzyme in nanomotor-based systems.

**Lipase** is a hydrolase enzyme that catalyzes the hydrolysis of fatty acid esters into glycerol and free fatty acids:

$$Triglyceride+3H_{2}O\to Glycerol+3\;Fatty\;Acids$$

The products generated during this reaction modify the interfacial tension and alter the local physicochemical environment. These changes induce autonomous motion through a self-diffusiophoretic mechanism. Due to its high affinity toward hydrophobic substrates, lipase is particularly effective in systems containing oils, fats, and lipid emulsions [47]. Wang et al. [48] demonstrated that lipase-coated mesoporous nanoparticles exhibited autonomous movement in triglyceride-containing media and significantly enhanced lipid degradation efficiency. Lipase-driven nanomotor systems have demonstrated an approximately 50% increase in the diffusion coefficient compared with passive Brownian motion. Owing to their ability to actively degrade lipid deposits and emulsions, lipase-based nanomotors have attracted considerable interest in biomedical applications. These systems may be utilized in the treatment of metabolic disorders, the removal of lipid microparticles from the vascular system, and the enhancement of drug transport in lipid-rich biological environments [35].

**Figure 2:**
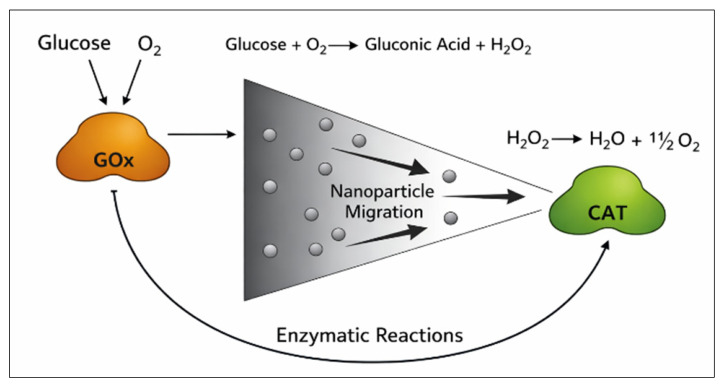
Schematic representation of the GOx–CAT enzymatic cascade. GOx catalyzes glucose oxidation to produce H_2_O_2_, which is subsequently decomposed by CAT into water and oxygen, promoting nanoparticle migration and improving the overall catalytic process. Abbreviations: GOx—glucose oxidase; CAT—catalase.

**Enzyme cascades** consist of sequential combinations of multiple enzymes, such as GOx and CAT [49] (Figure 2). They enable optimization of catalytic efficiency, reduction of toxic intermediates, and enhanced performance under physiological conditions. In addition, cascade systems can exhibit “smart” behavior, for example by modulating reaction rates in response to substrate concentration [33]. Their implementation also mitigates the accumulation of harmful by-products, such as H_2_O_2_, thereby improving the biocompatibility of enzymatic engines in biological environments [2].

### 2.2. ATP-Dependent Motor Proteins as Biological Nanomotors

ATP-dependent motor proteins, such as myosins, kinesins, and dyneins, are biological nanomotors that convert chemical energy derived from ATP hydrolysis into directed mechanical motion along cytoskeletal filaments. This process enables intracellular transport and force generation [50]. Unlike EMNMswhich rely on locally generated chemical gradients produced during catalytic reactions, motor proteins operate via a tightly coupled ATPase cycle and coordinated conformational changes that ensure processive, stepwise movement along their respective filaments.

**Myosins** are ATP-dependent motor proteins that move along actin filaments. They utilize the energy from ATP hydrolysis to generate stepwise conformational changes. The mechanism of action is based on a cycle of ATP binding, hydrolysis, and release, which translates into directional movement and the generation of mechanical force on the nanoscale. In bioengineering systems, myosins serve as a model example of the conversion of chemical energy into linear motion, providing inspiration for hybrid nanotransport devices [51]. These mechanisms have potential applications in medicine, particularly in the design of biomimetic drug delivery systems, biosensors, and molecular diagnostic systems based on motor proteins [52].

**Kinesins** are double-headed motor proteins that move processively along microtubules via coordinated, ATP-driven conformational changes. This enables a “hand-over-hand” stepping mechanism. Their function is characterized by high energy efficiency and the precise transport of intracellular cargo, including vesicles and organelles [53]. In nanomedicine, kinesin-based systems are being explored as biomimetic platforms for the targeted transport of therapeutic agents and diagnostic nanosystems within controlled microfluidic environments [54].

**Dyneins** are complex motor proteins responsible for movement toward the minus end of microtubules. They harness energy from ATP hydrolysis to generate substantial mechanical forces through conformational changes within their AAA+ (ATPases Associated with diverse cellular Activities) domains. Unlike kinesins, dyneins exhibit a more intricate stepping mechanism involving coordinated head communication and elastic structural rearrangements [55]. In biomimetic systems, dyneins serve as model platforms for asymmetric, high-force nanomotors.

### 2.3. Motion Mechanisms

**Figure 3:**
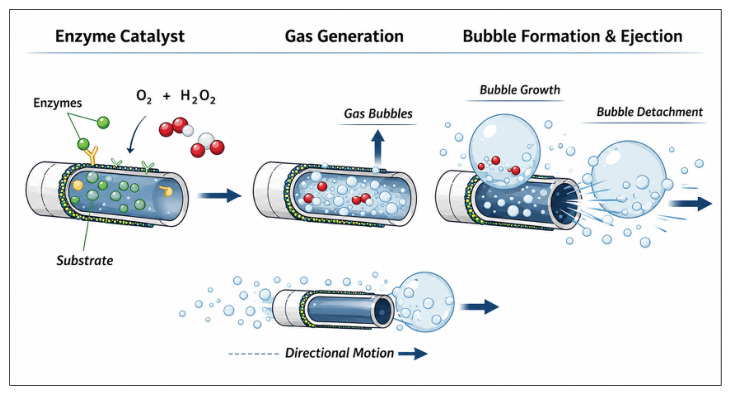
Bubble-driven propulsion of enzyme-powered micromotors. Enzymatic reactions produce gas bubbles that nucleate, grow, and detach from the micromotor, generating thrust and enabling directional movement.

Enzyme-driven micro- and nanostructures can generate motion through a range of physicochemical mechanisms, including self-diffusiophoresis, reaction-induced concentration gradients, and interfacial effects [56]. It has been demonstrated that catalytic substrate decomposition can produce asymmetric distributions of reaction products, which act as a primary driving force for many biocatalytic micromotors [57]. Other systems exploit mechanisms such as local viscosity changes, interfacial stress modulation, or gas bubble generation, enabling diverse propulsion modes and enhanced directional control [58,59]. The selection of enzyme, substrate availability, and particle architecture signifi-cantly influence performance metrics like speed, directional persistence, and re-sponsiveness to biological gradients. Nanomotor architecture is a key determinant of propulsion performance, as particle geometry, surface asymmetry, and enzyme distribution directly influence the formation of local concentration gradients that drive motion. Asymmetric designs, and spatially controlled enzyme immobilization, generally promote higher propulsion efficiency, improved directional persistence, and enhanced responsiveness to chemical gradients. Moreover, enzyme loading and catalytic configuration affect substrate conversion rates and consequently nanomotor velocity and navigation behavior in biological environments. Therefore, rational architectural design is essential for optimizing the performance of EMNMs in biomedical applications [35,39]. Enzymatic motion can be achieved through various physicochemical mechanisms:

**Bubble propulsion** in micromotors is based on the generation and expulsion of gas bubbles (e.g., H_2_, O_2_, CO_2_). The processes of nucleation, growth, and detachment from the surface of the structure produce a thrust force that drives motion in the surrounding fluid (Figure 3) [60,61]. Review studies on the physicochemical principles of bubble propulsion highlight that the key parameters governing performance include the frequency of bubble formation, bubble size, and their interactions with the motor surface. These factors collectively determine the speed and controllability of micromotor motion [62]. The efficiency of bubble propulsion is influenced by enzyme selection, as enzymes with high turnover numbers and the ability to produce gas bubbles are preferred. Particle design, such as tubular or asymmetric geometries, enhances bubble for-mation and detachment, leading to higher velocities. Recent research has focused on controlling bubble dynamics to improve motion precision and stability [26]. In addition, hybrid systems increasingly incorporate enzymes such as CAT or urease, which catalyze gas-generating reactions (e.g., O_2_ or CO_2_ production). This enables bubble propulsion under biologically benign conditions. The integration of enzymatic components with bubble-based micromotors enhances system biocompatibility and enables additional levels of motion control through the regulation of enzymatic activity and substrate availability [60,63].

**Figure 4:**
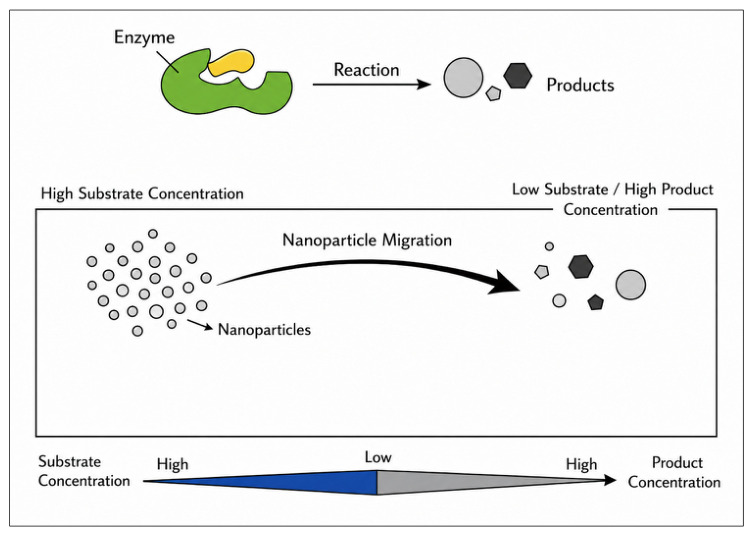
Diffusiophoresis and nanoparticle migration induced by enzymatic reactions. The enzymatic conversion of substrates into products establishes concentration gradients that generate diffusiophoretic forces, resulting in the directional migration of nanoparticles.

**Diffusiophoresis (chemophoresis)** is a mechanism in which a concentration gradient of a substrate or enzymatic reaction products induces the migration of nanoparticles (Figure 4). This process involves particle movement along a chemical gradient generated by surface interactions. It is driven by spatial variations in the chemical potential of the surrounding solution, without the need for external electric or hydrodynamic fields. In enzymatic systems, such gradients can arise locally as a consequence of catalytic reactions. These reactions produce asymmetric concentration profiles that drive the directional motion of enzyme-functionalized nanoparticles. Such phenomena have been described in models of self-propelled EMNMs [33]. In contrast, Shandilya et al. [64] reported a system in which catalytic microparticles in microchannels responded to adenine nucleotide gradients (AMP/ADP/ATP). Their motion was regulated by the enzymatic hydrolysis of ATP to AMP and Pi, catalyzed by potato apyrase. For instance, urease decomposes urea into ammonia and carbon dioxide, creating a pH gradient that drives motion. Particle architecture, including size and surface asymmetry, is crucial for establishing effective concentration gradients. Studies have shown that asymmetric catalyst localization is essential for generating the necessary gradients for propulsion [26].

**Chemotaxis:** Enzyme-powered motors can respond to substrate concentration gradi-ents, allowing preferential migration toward regions enriched in biofuel. This behavior enhances navigation in complex biological environments. The enzyme’s affinity for the substrate and the rate of product formation determine the gradient’s strength and di-rectionality. Particle architecture, including surface properties and symmetry, affects the motor’s responsiveness to these gradients. Studies have demonstrated that the in-terplay between phoretic effects and enhanced diffusion can influence chemotactic behavior, with the dominance of one mechanism over the other depending on sub-strate concentration [2,22,26].

**Figure 5:**
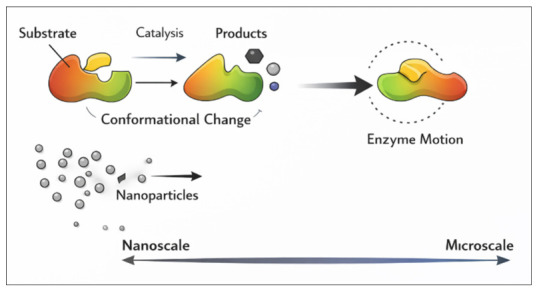
Schematic representation of enzyme conformational dynamics during catalysis. Substrate binding induces structural rearrangements within the enzyme, followed by product formation and release. Repeated catalytic cycles generate conformational fluctuations and local concentration gradients that may contribute to enhanced diffusion and autonomous motion. This phenomenon is considered one of the potential mechanisms involved in the propulsion of enzymatic nanomotors and nanoscale catalytic systems.

**Conformational dynamics** of enzymes represent a key mechanism potentially linking catalysis to motion generation at the nano- and microscale (Figure 5). Contemporary approaches describe enzymes as dynamic systems that undergo multiple conformational states with distinct free energy levels during the catalytic cycle. These states are associated with substrate binding, active-site reorganization, and product release [65]. Such nonequilibrium transitions may generate internal forces and mechanical fluctuations capable of interacting with the surrounding medium [66]. These non-equilibrium transitions can generate internal forces and mechanical fluctuations capable of interacting with the surrounding medium. Theoretical models of active enzymatic matter suggest that cyclic conformational changes lead to effective “pushing” of the surrounding fluid through asymmetric hydrodynamic deformations, resulting in enhanced diffusion [67]. More recent studies indicate that coupling between the enzyme’s energy landscape and conformational dynamics may enable the conversion of chemical energy into mechanical motion. This mechanism resembles the operation of a molecular fluctuation motor, particularly under non-equilibrium conditions. Molecular simulations and single-molecule experiments suggest that the amplitude and anisotropy of conformational changes are critical for the efficiency of this process. This implies that not all enzymes are capable of generating measurable propulsion. Recent reviews further emphasize that conformational effects likely coexist with diffusive and thermophoretic mechanisms. This results in a complex, multi-component picture of chemo-mechanical coupling in enzymatic systems [65,68].

**Small local changes** in charge distribution and temperature occur during enzymatic reactions, which can influence particle motion in solution. Reaction products generate local electrochemical and thermal gradients that induce electrokinetic and thermophoretic effects, leading to directional particle migration or increased effective diffusion. This is consistent with observations of enhanced diffusion and enzyme drift in substrate gradients [69]. The energy released during enzymatic catalysis can also induce fluid flows and particle transport in the vicinity of enzymes. Sapre et al. [70] investigated enzymes immobilized on supported lipid bilayers (SLBs). It has been demonstrated that catalytic activity generates kinetic flow within the fluid. This flow is responsible for the observed motility and directional transport of tracer particles on lipid surfaces. Enzymatic catalysis, as well as thermal diffusion processes, induce characteristic motion and transport within two-dimensional membrane environments. This highlights the active, far-from-equilibrium effects generated by enzymes during catalytic reactions [70].

A comparison of enzymatic propulsion mechanisms suggests that diffusiophoresis is more effective in low-viscosity environments with small substrate gradients, whereas bubble propulsion dominates in vitro under high substrate concentration conditions. To facilitate comparison of the major enzymatic propulsion strategies discussed above, Table 1 summarises the key characteristics of representative enzymatic nanomotor systems.

The interplay between enzyme selection, substrate availability, and particle architecture is critical in optimizing the performance of enzyme-powered micro/nanomotors. Tailoring these factors allows for the design of motors with desired propulsion characteristics, enhancing their applicability in biomedical and environmental applications. A comparison of enzymatic propulsion mechanisms suggests that diffusiophoresis is more effective in low-viscosity environments with small substrate gradients, whereas bubble propulsion dominates in vitro under high substrate concentration conditions.

## 3. Design of Enzymatic Nanomotors (EMNMs)

The design of enzymatic nanomotors (EMNMs) plays a pivotal role in determining their propulsion performance, stability, controllability, and biomedical functionality. Key design parameters, including scaffold materials, particle size and shape, enzyme immobilization strategies, and motion control mechanisms, collectively influence catalytic efficiency, movement behavior, and interactions with biological environments. Understanding the relationship between these structural and functional elements is therefore essential for the rational development of EMNMs with enhanced performance for diagnostic and therapeutic applications.

### 3.1. Scaffold Materials

Scaffold materials play a crucial role in the design of EMNMs, determining their stability, biocompatibility, and catalytic efficiency. Synthetic polymers and biopolymers enable stable enzyme immobilization and surface functionalization [26]. Inorganic structures, including mesoporous silica, allow enzyme encapsulation and controlled interactions with substrates. Metal–organic frameworks (MOFs) protect enzymes from denaturation while preserving their catalytic activity [71]. Thus, the choice of scaffold material directly influences both enzyme immobilization and the self-propulsion mechanisms of nanomotors.

**Biodegradable polymers (PLGA, chitosan)** are widely used in EMNMs due to their high biocompatibility and controlled degradation within the body [72]. These polymers can be functionalized with enzymes (e.g., CAT or urease) immobilized on their surface or within the structure. In this configuration, they catalyze the decomposition of biofuels such as H_2_O_2_ or urea, generating chemical gradients that drive self-propulsion. The use of biodegradable materials also reduces the risk of long-term accumulation of nanostructures in tissues, which is essential for clinical translation. For example, biodegradable PLGA micromotors with asymmetric enzyme distribution exhibit chemotactic behavior toward inflammatory sites. They also show active motion in biological environments, supporting targeted drug delivery applications.

**Mesoporous silica (MSNs)** is widely used in the design of EMNMs due to their large surface area, ease of chemical functionalization, and high loading capacity for both drugs and enzymes. MSNs act as a structural scaffold enabling the immobilization of enzymatic catalytic units (e.g., urease) while simultaneously encapsulating therapeutic molecules. They also provide controlled, stimulus-responsive release [72]. Consequently, MSN-based nanomotors can exhibit enhanced diffusion in the presence of substrate. They also enable on-demand drug release, which is highly relevant for advanced drug delivery systems. In cellular models, it has been shown that enzymatic activity and propulsion of MSN-based nanomotors enhance cellular uptake and promote payload release within acidic intracellular vesicles.

**Liposomes and lipid nanoparticles** provide a versatile platform for the development of EMNMs due to their biocompatibility, structural flexibility, and ability to incorporate functional enzymes. In addition, they enable the integration of hydrodynamic and interfacial features that support motion and functional activity. These lipid-based systems can be engineered as EMNMs, where local substrate conversion by enzymes anchored in the lipid bilayer or encapsulated within the vesicle generates propulsive forces. Liposomes can also serve as carriers for therapeutic payloads and enable their controlled release at target sites, making them highly attractive platforms in nanomedicine [73]. Experimental studies on enzymatic liposomal nanomotors powered by electrolytically generated gas have demonstrated that lipid nanostructures are capable of directional motion in aqueous environments. These findings represent an important step toward therapeutic and targeted drug delivery applications.

**Metals (Au, Pt) in hybrid systems.** Metals such as Au and Pt are frequently incorporated into EMNMs to combine the catalytic properties of metals with enzymatic biocatalysis [74]. These metal components can catalyze substrate decomposition (e.g., H_2_O_2_) and introduce structural asymmetry characteristic of Janus systems, thereby promoting chemical propulsion and directional motion. Metal surfaces also enable straightforward functionalization with enzymes, ligands, and sensing molecules, increasing the multifunctionality of the system. Hybrid Pt–MSN nanomotors with asymmetric architectures have demonstrated rapid self-propelled motion driven by fuel decomposition and show potential for enhanced biological penetration and intelligent drug delivery [75].

The choice of material depends on the intended biomedical application. Polymers and liposomes provide high biocompatibility; silica enables precise control over structure and porosity, whereas metallic components allow additional magnetic and optical modulation of nanomotor activity.

### 3.2. Size and Shape

Size is a key parameter affecting the efficiency of EMNMs. It influences both motility and penetration through biological barriers. Structures smaller than 200–300 nm show enhanced transport across the endothelium, extracellular matrix, and tissue microchannels [76]. Recent studies indicate a strong trend toward miniaturization due to the growing importance of biomedical applications within the human body [26]. Sun et al. [77] developed ultra-small stomatocyte nanomotors (USSNs) with diameters of ~150 nm using an extrusion method. These nanomotors showed enhanced accumulation at target sites and improved delivery efficiency compared with larger nanocarriers. Actively propelled nanomotors can overcome biological barriers more effectively than conventional systems. This results in deeper tissue penetration and higher cellular internalization. Geometry (stomatocytic, spherical, cylindrical) is also a key factor. It affects interactions with the environment and the efficiency of converting enzymatic energy into motion. This has been confirmed for CAT- and urease-based nanomotors [26,78]. Active transport mechanisms are particularly important for cellular processes, including the delivery of bioactive molecules to intracellular structures. This is also relevant in plant systems, where efficient metabolite transport determines biological activity and therapeutic potential [79,80].

### 3.3. Enzyme Immobilization

In modern enzymatic nanomotor systems, the choice of strategy (immobilization, encapsulation, or surface functionalization) determines both stability and catalytic efficiency [81]. This is particularly relevant in biological environments enriched with plant-derived compounds. Enzyme encapsulation within nanostructures (e.g., metal–organic frameworks or polymeric nanocarriers) creates a protective microenvironment. This reduces enzymatic degradation and improves biocompatibility. It also enables controlled activity in the presence of reactive plant metabolites, such as polyphenols and terpenes [82]. Surface functionalization increases the accessibility of active sites and enhances substrate–enzyme interactions. This has been demonstrated for enzymes immobilized on magnetic nanoparticles and carbon-based materials. Both covalent and non-covalent interactions contributed to protein stabilization and improved catalytic efficiency [83]. In enzyme-driven nanomotors, such approaches enable the use of plant extracts as both substrate sources (e.g., H_2_O_2_ generated by plant oxidases) and bioactive modulators of the reaction environment. This leads to “smart” systems with adaptive and selective behavior in therapeutic and environmental applications [35]. The integration of enzymes with nanocarriers inspired by plant-derived biopolymers (e.g., chitosan, lignin) further enhances system stability, biodegradability, and clinical potential. This highlights the increasing role of plant-based materials in the design of future biohybrid nanomotors [84].

### 3.4. Motion Control and “Swarms”

In addition to self-propulsion (i.e., autonomous motion without external mechanical input), directional control of microcarriers and nanomotors is a key functional requirement in biomedical applications. In chemotaxis-based systems, motion can be precisely modulated by local gradients in reagent concentration. This enables autonomous migration toward regions with specific chemical signatures. These mechanisms constitute the basis of bio-inspired navigation and are actively developed in synthetic micro- and nanorobotic systems [85]. Importantly, these approaches can be combined with natural herbal extracts. Due to their bioactive compounds (e.g., alkaloids, flavonoids, and essential oils), they can generate local chemical gradients. These gradients may act as guiding signals or as a source of “biofuel” in biohybrid systems [86]. In magnetic control strategies, external magnetic fields enable contactless actuation and deep penetration into biological environments. They also allow precise regulation of motion trajectories. As a result, magnetic guidance is one of the most widely used approaches in micro- and nanorobotic systems. Magnetically activated structures can be controlled by magnetic forces and torques. This enables complex motion patterns in fluid environments [87]. In contrast, optical control relies on photosensitive materials that respond to light by changing their physicochemical properties. This induces local propulsion asymmetry and enables motion control. This approach provides high spatial resolution and allows real-time programming of trajectories, particularly in systems based on optically responsive nanomaterials [88]. Natural plant extracts, such as curcumin (a polyphenol) and soy isoflavones (flavonoids including genistein and daidzein), can act as bioactive photosensitizers and modulators of the redox environment in photoreactive systems [37]. Upon light absorption, these compounds enter an excited state. This can lead to the generation of reactive oxygen species (ROS) or shifts in redox potential. As a result, local modification of material properties occurs, enabling control over physicochemical responses [81]. In this context, plant metabolites can be integrated with photostable micro- and nanostructured systems [89,90]. The coordinated swarming of micro- and nanomotors significantly enhances their transport efficiency. It also enables complex therapeutic functions through the transition from individual behavior to emergent collective systems. In enzymatic systems, swarms can form spontaneously due to chemical gradients and hydrodynamic interactions. This leads to improved penetration of biological environments and enhanced cargo transport efficiency. Such systems exhibit multitasking and functional synergy, which markedly exceeds the performance of individual nanomotors [22]. A key advantage is the use of biocompatible fuels, such as glucose or urea, enabling in vivo operation without toxic substrates [2]. Swarming further amplifies local mechanical and chemical effects. This can be exploited for targeted drug delivery and enzymatic therapy. An important extension of these systems is their integration with bioactive plant extracts, including cereal-derived secondary metabolites (e.g., ferulic acid, flavonoids, phytosterols). These compounds can act both as therapeutic payloads and as surface modifiers for nanostructures [91]. Such biohybrid systems pave the way for “smart therapeutic swarms” that integrate enzymatic activity with the bioactivity of phytochemicals. Consequently, the synergistic combination of enzyme-driven swarms and plant-derived compounds represents a promising platform for advanced drug delivery systems. These systems can adapt to the disease microenvironment and exhibit enhanced therapeutic efficacy in vivo [92].

The design of enzymatic nanomotors is influenced by multiple structural and function-al parameters that collectively determine their propulsion efficiency, stability, and bi-omedical performance. The major design considerations are summarized in Table 2.

## 4. Plant Extracts as Functional Modulators

In addition to primary metabolites, plants synthesize a wide range of secondary metabolites, including polyphenols (e.g., flavonoids), alkaloids, terpenoids, and saponins. These compounds exhibit significant bioactive potential and are widely present in medicinal and agricultural plants used for food, feed, and industrial applications [93,94]. Secondary metabolites play important roles in plant communities within ecosystems and agroecosystems. They also represent valuable resources for the food and pharmaceutical industries [95]. Industrial-scale raw materials are obtained not only from natural habitats but primarily from medicinal and agricultural crops. High-quality plant material is ensured by Good Agricultural Practice (GAP) and by the implementation of modern technologies and machinery aligned with smart, carbon-neutral, and environmentally sustainable agriculture [96,97]. They are developed, among others, as part of projects co-financed by the European Union promoting sustainable development and agriculture 4.0 [98,99]. The biological activity of plant extracts often results from the synergistic action of multiple secondary metabolites present within plant tissues [95]. These compounds exhibit antioxidant, anti-inflammatory, antimicrobial, and anticancer properties [100]. Phenolic compounds and flavonoids represent a particularly important group due to their strong antioxidant and anti-inflammatory activities. These effects are largely associated with their ability to neutralize reactive oxygen species and reduce oxidative stress [37,101]. Many secondary metabolites present in plant extracts also exhibit anticancer activity. These compounds can suppress tumor progression by inhibiting cancer cell growth and proliferation [101]. Phenolic compounds, essential oils, and terpenoids also demonstrate antibacterial activity against both Gram-positive and Gram-negative bacteria, highlighting their importance in pharmaceutical and biomedical applications [100]. Despite their high biological potential, the technological and clinical application of plant extracts remains limited by several factors. A major challenge is the variability in the qualitative and quantitative composition of secondary metabolites. This variability depends on plant species, environmental conditions, cultivation practices, harvest time, and extraction or storage methods. In addition, many bioactive compounds exhibit limited physicochemical stability and may degrade under exposure to light, temperature, or humidity [102]. The bioavailability of plant-derived secondary metabolites depends on their chemical structure as well as digestion and metabolic processes. Compounds such as polyphenols, flavonoids, and carotenoids often exhibit low bioavailability due to poor solubility and transformations occurring in the gastrointestinal tract [103]. These limitations can be partially overcome through nanotechnology-based approaches that improve the stability and bioavailability of plant-derived compounds [104].

## 5. Synergistic Integration: Enzymatic Nanomotors + Plant Extracts

The integration of enzymatic nanomotors (EMNMs) with plant-derived extracts represents a promising strategy for enhancing the therapeutic and biomedical capabilities of these nanosystems. By combining the active propulsion of EMNMs with the diverse biological activities of phytochemicals, such hybrid platforms can achieve improved cargo delivery, increased bioavailability of bioactive compounds, and enhanced therapeutic efficacy. The design of integration strategies, the underlying mechanisms of synergy, and the resulting biointeractions are therefore critical factors determining the overall performance and potential applications of EMNM–plant extract systems.

### 5.1. Integration Strategies

A detailed analysis of enzyme immobilization on nanocarriers containing plant extracts indicates that physical adsorption, cross-linking, and covalent attachment are the predominant strategies. These approaches aim to spatially stabilize the catalytic protein and optimize its local microenvironment [105]. Secondary metabolites, particularly polyphenols, exhibit a strong ability to form hydrogen and electrostatic interactions with biomolecules. Notably, approximately 63% of nanomotors rely on covalent enzyme–carrier conjugation, which enhances structural stability but may, in some cases, reduce catalytic efficiency [26]. In encapsulation strategies, the core-shell architecture is the most commonly used approach. This system provides optimal conditions for enzymatic activity while protecting enzymes from the external environment and premature degradation. At the same time, the modified surface maintains direct interaction with endogenous substrates. Following delivery to target tissues, the carrier undergoes controlled biodegradable degradation [106]. Chemical coupling remains one of the most advanced methods for integrating nanomotor components. This approach enables the formation of stable covalent bonds through the direct linkage of functional groups derived from plant extracts. As a result, premature drug release can be significantly reduced [107]. Chemical coupling has also been applied in the development of nanocarriers capable of selectively targeting cancer cells, including CHO and SK-BR-3 cell lines, in vitro. The materials used for the synthesis of these hybrid nanostructures are considered relatively safe and suitable for clinical applications [108]. The synergistic integration of EMNMs with bioactive plant extracts represents a promising strategy for advanced drug delivery systems. Enzyme-driven nanomotors exhibit efficient transport and enhanced penetration of biological environments [109]. In contrast, plant extracts provide secondary metabolites with diverse biological activities [110]. Major integration strategies include enzyme immobilization, encapsulation, and chemical coupling. Immobilization improves enzyme stability and catalytic activity [111], whereas encapsulation enables protection and controlled release of active compounds [26]. The use of plant extracts is also consistent with green synthesis approaches and may enhance system biocompatibility [112]. These hybrid systems show strong potential in biomedical applications, particularly in targeted therapy and controlled drug delivery.

### 5.2. Mechanisms of Synergy

The integration of enzyme-driven systems with bioactive plant extracts offers unique opportunities for functional synergy in advanced nanocarrier design. Effective targeting of tumor tissues requires precise and active transport mechanisms. Long-term studies indicate that only approximately 0.7% of conventional nanoparticles reach solid tumor sites [113]. The integration of biocatalytic systems utilizing endogenous fuels enables the conversion of chemical energy into kinetic energy. This process drives autonomous motion, referred to as self-propulsion. The resulting active thrust enhances deep penetration of nanocarriers into the tumor microenvironment [26]. A major challenge in the biomedical application of nanomotors is the risk of excessive oxidative stress generated during propulsion reactions. In this context, phytochemicals such as polyphenols and flavonoids may provide protective effects due to their natural reactive oxygen species scavenging activity. The antioxidant shield provided by these compounds enables continuous neutralization of excess cytotoxic byproducts, thereby protecting both enzymes and healthy host cells [2,114].

### 5.3. Biointeractions

A comprehensive understanding of hybrid nanocarriers in therapeutic applications requires detailed analysis of their interactions with target cells and the pathological microenvironment. Active endocytosis remains the primary mechanism of cellular internalization for these advanced systems [114]. Autonomous propulsion of nanomotors, driven by oxygen gradients generated via catalytic decomposition of hydrogen peroxide, increases the probability of contact between carriers and surface receptors. It also facilitates penetration of the cellular glycocalyx barrier. A major intracellular limitation of such systems is lysosomal degradation. To overcome this, self-propulsion strategies based on CaCO_3_ decomposition have been developed. This process induces a proton-sponge-like effect and triggers rapid CO_2_ release. The resulting vesicle destabilization enables efficient escape of nanocarriers into the cytoplasm [115]. The solid tumor microenvironment is characterized by specific physiological abnormalities, such as elevated interstitial pressure and a dense extracellular matrix [113]. Other significant features include unique acidity (pH 6.2–6.8) and excessive levels of reactive oxygen species (ROS). These parameters can be successfully utilized as triggers for therapy. The characteristic reduction in pH enables the intelligent and gradual release of the payload into the solid tumor, bypassing healthy tissues [106]. In turn, reactive oxygen species are broken down by CAT present in the nanomotors, providing propulsive drive while simultaneously protecting the patient’s healthy cells from oxidative stress [114].

## 6. Biomedical Applications

EMNMs are promising tools for drug delivery and diagnostics. Their autonomous motion enables active penetration of biological tissues. They are increasingly explored in cancer therapy. For example, collagenase-powered nanomotors degrade collagen in the extracellular matrix. This remodels the tumor microenvironment and enhances drug penetration and efficacy [35]. In another approach, amine oxidase-powered nanomotors use tumor-associated polyamines as fuel. This generates toxic byproducts and suppresses tumor cell growth [116]. In addition, EMNMs have been applied in other medical areas, including the gastrointestinal tract. They enhance delivery to the intestinal epithelium and improve the efficacy of oral therapies [117]. Another important application is the treatment of bacterial infections. Nanomotors can efficiently penetrate biofilms and increase contact with pathogens, leading to improved antimicrobial efficacy [118]. Nanomotors have also been investigated in autoimmune diseases, such as rheumatoid arthritis. Hydrogen peroxide-activated systems generate oxygen inflamed tissues. This reduces oxidative stress and alleviates inflammation and joint damage [119]. These systems have also been used as biosensors in diagnostics, enabling the detection of nucleic acids, cancer cells, bacteria, and proteins [120]. CAT-based systems represent one example, using hydrogen peroxide as a fuel source. Their application in the rapid detection of procalcitonin (PCT), a sepsis biomarker, has been demonstrated. Nanomotor motion is converted into an analytical signal, enabling rapid readout of results. The analysis time was significantly shorter than that of conventional methods. These systems can be easily modified by changing the recognition elements, enabling detection of a wide range of biomarkers [35]. There is also growing interest in nanomaterials synthesized using plant extracts rich in bioactive metabolites. These metabolites contribute to the stabilization and functionalization of nanostructures. Particular importance is attributed to plants of the genus Annona, whose extracts have demonstrated antimicrobial, antifungal, and antiparasitic activities. These include effects against mosquito larvae responsible for transmitting diseases such as dengue, Zika, and chikungunya [104].

## 7. Safety, Toxicity, and Regulatory Issues: Challenges and Limitations

Despite their promising therapeutic potential, enzyme–nanocarrier systems are associated with several potential risks and side effects. These require thorough evaluation prior to clinical application. For example, metallic or non-biodegradable nanoparticles may exhibit intrinsic toxicity independent of the loaded therapeutic agent. Toxicological reviews indicate that different types of nanocarriers (metallic, lipid, and protein-based) may induce oxidative stress, DNA damage, and hepatotoxic and nephrotoxic effects [121]. Non-biodegradable nanoparticles, in particular, tend to accumulate in the liver, spleen, and kidneys [122]. The immunogenicity of enzymes and other therapeutic proteins remains a key challenge in their development, as it can lead to the formation of anti-drug antibodies, reduced therapeutic efficacy, and hypersensitivity reactions [123]. This risk is particularly significant for non-human or modified enzymes, such as PEGylated forms. Immune responses may still occur in these cases despite structural and formulation-based mitigation strategies. In addition, safety concerns extend beyond immunogenicity and include protein instability, aggregation, and potential adverse effects due to off-target biological activity. These factors complicate the toxicological and regulatory assessment of such preparations. Consequently, multi-step preclinical and clinical evaluation strategies are required. Approaches aimed at inducing immunological tolerance are also being explored, although their clinical effectiveness remains limited [124]. At the same time, it is emphasized that despite advances in protein engineering (e.g., PEGylation and humanization), immune responses cannot be fully eliminated. This necessitates further development of risk mitigation and safety monitoring strategies. A detailed analysis of enzyme immunogenicity indicates that the main barrier to the therapeutic use of nanocarriers is the formation of anti-drug antibodies (ADAs). These antibodies can exhibit neutralizing activity, directly blocking active sites and inhibiting catalytic function [125]. In addition, the phenomenon of accelerated blood clearance (ABC) significantly limits the feasibility of repeated administration of the same nanocarriers. This complication is particularly evident in the surface modification of nanocarriers with polyethylene glycol (PEG). PEG modification is intended to confer “stealth” properties and reduce immune recognition. However, studies have shown that a second dose of PEGylated enzymes or liposomes can be rapidly cleared from circulation, particularly when administered within a specific time window after the initial exposure [126]. Another important aspect of nanomedicine development is the toxicity of nanocarriers and their biodistribution in the human body. Global biodistribution studies indicate that up to 99% of nanocarriers are sequestered in filtering organs before reaching target tissues. They are taken up by macrophages; however, due to the lack of biodegradability of synthetic cores, they cannot be enzymatically degraded. As a result, these particles can accumulate in the body and induce oxidative stress, inflammation, and organ dysfunction [113]. Furthermore, specific physicochemical properties of nanocarriers may trigger premature activation of the complement system, leading to an immediate systemic pseudoallergic reaction (CARPA). This phenomenon can manifest as acute hemodynamic disturbances, bronchospasm, or anaphylactoid shock. This represents a significant technological and biological barrier that limits the direct clinical translation of advanced nanocarriers [127].

Due to the complex composition of plant extract–based preparations, ensuring consistent quality and safety remains a major challenge [128]. An additional limitation of enzyme–plant extract formulations is the difficulty in achieving high reproducibility of phytochemical composition. This variability complicates the assessment of product quality and safety. According to FDA guidelines, botanical products require strict quality control and batch-to-batch consistency monitoring due to their complex and heterogeneous composition [129]. The EMA emphasizes the need for accurate identification of plant material, standardization of manufacturing processes, and the use of chromatographic methods and quality markers to ensure reproducibility of herbal preparations [130]. Attention is also drawn to the control of contaminants, including pesticides, heavy metals, mycotoxins, and microbiological impurities [129,130].

## 8. Clinical Perspectives and Translation

From a clinical perspective, the main advantage of enzymatic nanomotors is their ability to move actively within biological environments and to respond to disease-specific biochemical stimuli [105]. These properties can significantly improve tissue penetration, local drug retention and therapeutic precision compared to conventional passive nanocarriers [113]. The integration of plant-derived bioactive compounds with enzyme-powered nanocarriers has emerged as a particularly promising strategy due to the possibility of combining active transport mechanisms with the antioxidant, anti-inflammatory, and anticancer properties of phytochemicals [2]. Recent studies have demonstrated enhanced tumor penetration, improved blood–brain barrier transport, biofilm disruption, ROS-responsive therapeutic delivery, and accelerated tissue repair using these multifunctional systems. Such multifunctional systems have the potential to generate new opportunities for personalised and targeted therapies in oncology, neurology, inflammatory diseases, and regenerative medicine [131].

The representative studies summarised in Table 3 demonstrate that enzymatic nanomotors and phytochemical-based nanosystems can improve tumour penetration, targeted drug delivery and therapeutic efficacy. These approaches have shown promising anti-tumour effects through mechanisms including chemotherapy, starvation therapy, photodynamic therapy and immunomodulation. However, further research is needed to address biosafety and translational challenges.

Recent studies have demonstrated enhanced blood–brain barrier transport, reduced neuroin-flammation, and improved neuroprotection using curcumin-, flavonoid-, and nanomo-tor-based delivery systems. Potential translational applications in neurological disorders are outlined in Table 4.

Enzymatic nanomotors and phytochemical-based nanosystems have considerable potential for treating inflammatory diseases by regulating ROS and delivering therapeutics more precisely. Recent studies have demonstrated reduced oxidative stress, the suppression of pro-inflammatory signalling pathways and improved therapeutic outcomes in experimental models of inflammatory disorders. Representative approaches are outlined in Table 5.

Recent studies suggest that enzymatic nanomotors could support regenerative medicine applications by improving tissue penetration and localised therapeutic retention within damaged tissues and chronic wound microenvironments, as well as enabling ROS-responsive delivery. Furthermore, preclinical models have demonstrated that polyphenol-loaded nanocarriers and enzyme-responsive nanosystems can improve wound healing, reduce oxidative stress and enhance tissue regeneration. Representative strategies are summarised in Table 6.

**Figure 6:**
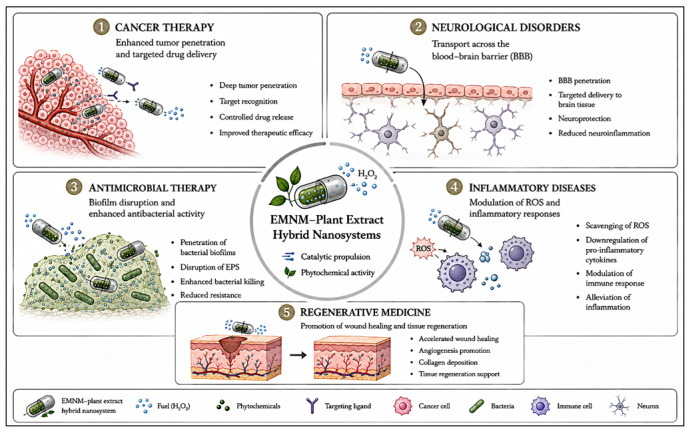
Disease-specific biomedical applications of enzymatic nanomotor–phytochemical hybrid nanosystems.

Figure 6 summarizes the principal therapeutic applications of enzymatic nanomotor–phytochemical hybrid (EMNMs) in diverse pathological environments. By integrating catalytic self-propulsion with the biological activity of plant-derived phytochemicals, these platforms enhance transport across biological barriers, improve target-site accumulation, and increase therapeutic efficacy. In oncology, EMNMs facilitate deep tumor penetration and targeted drug delivery; in neurological disorders, they enable transport across the blood–brain barrier; in antimicrobial therapy, they promote biofilm disruption and enhanced pathogen eradication; while in inflammatory and regenerative conditions, they modulate oxidative stress and inflammatory responses, thereby supporting tissue repair and regeneration.

The analysis of the strategies employed indicates that the utilisation of enzymatic nanomotors has the potential to markedly enhance the efficacy of phytochemical-based therapeutic interventions. This enhancement is attributed to the nanomotors’ ability to improve penetration of tumours, biofilms, inflamed tissues and damaged regenerative microenvironments. Representative studies have demonstrated enhanced tumour penetration, improved blood–brain barrier transport, suppression of inflammatory signalling pathways and accelerated wound healing through active, stimuli-responsive delivery mechanisms. ROS-responsive and biohybrid systems in particular demonstrate promising potential for targeted and controlled therapeutic delivery. However, further in vivo validation and long-term safety studies are essential before these systems can be used in clinical practice.

## 9. Development Prospects

The development of EMNMs is currently focused on improving their efficiency and biological safety [125]. As indicated in the study by Zhao et al. [145], there has been significant progress in the development of systems that utilise more than one enzyme. Enzyme cascade systems enhance the efficiency of nanomotor movement through the collaborative action of multiple enzymes. In such systems, intermediate products of one reaction are utilised in subsequent stages of the enzymatic process. This facilitates the more efficient utilisation of chemical energy for propulsion. As posited by the authors, these systems have been demonstrated to reduce the accumulation of hydrogen peroxide and other toxic intermediates, thereby enhancing the biological safety of the system [146]. The employment of multiple enzymes has been demonstrated to enhance the mobility of nanomotors, thereby facilitating their operation within complex biological microenvironments, including cancerous tissue and the milieu associated with diabetes [146]. Another area of development is that of “swarm” EMNMs. These systems utilise the interaction of multiple nanomotors within a single system. A plethora of studies have indicated that they exhibit the ability to self-organise, co-locate, and respond to changes in the biological environment [147]. A subsequent developmental step could involve the utilisation of chemical communication between them. Enzymatic systems capable of transmitting chemical signals between different groups of nanomotors have been described. In the future, this could enable the localisation of disease sites and the targeting of nanomotors transporting therapeutic substances to those sites [148]. A significant challenge in the further development of nanomotors is the assurance of their biodegradability and clinical safety. Following the completion of their designated function, the nanomotors must be safely extracted from the body without causing any deleterious side effects. In the future, there will be an increased utilisation of substrates that are naturally present in the body in the design of EMNMs. It is also possible that future research will encompass the development of nanomotors based on biodegradable polymers, such as PEG-b-PCL or PEG-b-PDLLA. These polymers have been observed to undergo a process of gradual degradation within the body following the completion of their intended function [125]. Computer modelling and simulation represent pivotal elements in the ongoing enhancement of nanosystems. These methodologies facilitate the conceptualisation of increasingly efficient systems and the prediction of their behaviour within biological environments [149]. Computer simulations have been shown to reduce the necessity for experimental research in some cases [150]. Another significant area of research is the development of methods for the external and internal control of the movement of nanosystems in a biological environment. One such example is the Janus model, which possesses an asymmetric structure that enables the control of both the propulsion method and the direction of movement. The control of these systems can be facilitated through the utilisation of a magnetic field, light, ultrasound, or an electric field [151]. Research also describes internal control systems, in which nanosystems respond to chemical gradients present in the biological environment [148].

A significant challenge in the clinical translation of enzymatic nanotechnology is the determination of biodistribution and immunogenicity of the designed systems. Notwithstanding the favourable outcomes emanating from research conducted on circulation time and cellular integration, which corroborate the biocompatibility and malleability of nanosystems, there persists a paucity of comprehensive design and control frameworks for their movement within the body [150,151,152]. The precise and targeted delivery of pharmaceuticals necessitates the delineation of the toxicological profile and the specific impact of nanosystems on the homeostasis of healthy tissues. It is only through a comprehensive understanding of these interactions that an accurate evaluation of the clinical effectiveness of nanosystems can be made, and consequently, the standardisation of industrial-scale production [153]. Another significant facet of biomedical nanosystem applications pertains to the conceptualisation of intelligent nanorobots that possess the capability to circumvent physical impediments and autonomously respond to biological signals. Presently, specialists from across the globe are engaged in interdisciplinary research, debates and initiatives with the objective of developing nanorobots that possess considerable translational potential. Concurrently, vigorous efforts are being undertaken to adapt the strategy of their operations to the complexity of actual clinical scenarios [154]. In the domain of nanotechnology, the integration of artificial intelligence represents a particularly significant development. The field of technology has the potential to significantly accelerate the discovery of low-toxicity nanomaterials, as well as to facilitate the prediction of interactions between drugs, nanostructures, and biological systems [155,156,157]. At the micro-scale, the apparatus can be utilised for the purpose of active control of movement and planning of optimal pathways in complex tissue environments. Notwithstanding the technological challenges, including the necessity of miniaturising computational components, the execution of these designs remains a futuristic undertaking [158]. The advancement of nanotechnology is indicative of a trajectory that is orientated towards the realms of personalised medicine. The optimisation of nanorobot construction, the effective improvement of targeting using patient-specific data, and the facilitation of monitoring during treatment have the potential to lead to a paradigm shift in precision medicine [145]. A pivotal element of this theoretical framework is the integration of multi-enzymatic systems, which facilitate the execution of intricate and self-regulating catalytic processes. The synergistic action of enzyme cascades has been demonstrated to enhance the efficiency of the processes concerned. Furthermore, the presence of tandem arrangements (e.g., GOx) ensures effective regulation of the kinetics, as well as neutralisation of the toxic by-products. The objective is to establish a realistic and effective therapy for cancer that is both safe and effective [159].

Furthermore, the synergistic effects of such systems may be enhanced by biocomponents derived from plant extracts. The phytochemicals present in these extracts exhibit antioxidant and stabilising properties, thereby reducing oxidative stress and improving the biocompatibility and functional stability of nanotechnological platforms [159,160,161].

## 10. Conclusions

The latest advances in the field of enzymatic nanomotors were analyzed. The literature review indicates that this technology is rapidly progressing from fundamental research toward promising practical applications. Particular attention has been given to the use of natural plant extracts in the design and operation of such systems. Synergistic interplay between nanomotor-driven mobility and phytochemical bioactivity is a promising strategy for the development of next-generation precision therapeutics. Enzymatic nanomotors represent a particularly promising class of bioinspired nanosystems, with potential applications in targeted therapy, diagnostics, and precision medicine. It is hypothesized that their active propulsion and responsiveness to biological stimuli may improve drug delivery efficiency in comparison with conventional passive nanocarriers. Despite the encouraging preclinical results, there are still important challenges to be addressed, including biosafety, biodegradability, motion control, and large-scale reproducibility.

## Figures and Tables

**Table 1:** Comparison of major enzymatic propulsion systems used in nanomotors.

Enzyme/ System	Substrate/ Fuel	Main Reaction Products	Propulsion Mechanism	Advantages	Limitations
Catalase	H_2_O_2_	H_2_O, O_2_	Bubble propulsion and self-diffusiophoresis via oxygen generation	High catalytic efficiency, rapid propulsion, and good biocompatibility	Dependence on H_2_O_2_ and potential oxidative toxicity in vivo
Peroxidase	H_2_O_2_ and electron donors	Oxidized products and H_2_O	Diffusiophoresis driven by asymmetric substrate and product gradients	Responsive to local chemical gradients and ROS-rich microenvironments	Dependence on substrate availability and relatively lower propulsion efficiency
Urease	Urea	NH_3_, CO_2_	Self-diffusiophoresis generated by ionic and concentration gradients	Utilizes endogenous fuel, high biocompatibility, and good catalytic activity	Local pH alterations due to ammonia generation
Glucose oxidase (GOx)	Glucose and O_2_	Gluconic acid and H_2_O_2_	Diffusiophoretic propulsion and cascade-assisted oxygen generation	Exploits endogenous glucose and is suitable for enzyme cascade systems	Dependence on oxygen availability and limited use as a stand-alone propulsion system
Lipase	Triglycerides and lipids	Glycerol and free fatty acids	Self-diffusiophoresis associated with lipid hydrolysis	Effective in lipid-rich environments and compatible with biological substrates	Limited experimental validation compared with catalase- and urease-based systems
Enzyme cascade systems	Multiple endogenous substrates	Sequential reaction products	Cascade-enhanced propulsion and chemotaxis	Improved propulsion efficiency, amplified catalytic activity, and reduced toxic intermediates	Increased design and manufacturing complexity
ATP—dependent motor proteins	ATP	ADP + Pi	TP-driven conformational changes and filament-guided motion	Highly efficient and precise biological transport	Limited stability and applicability as synthetic nanomotors

**Table 2:** Major design parameters influencing the performance of enzymatic nanomotors.

Design Parameter	Representative Examples	Influence on Nanomotor Performance	Advantages	Limitations
Scaffold materials	PLGA, chitosan, mesoporous silica nanoparticles (MSNs), liposomes, lipid nanoparticles, MOFs, Au/Pt hybrid systems	Determine enzyme stability, catalytic activity, cargo loading, and propulsion efficiency	High biocompatibility, controlled drug release, enzyme protection	Material-dependent biodegradability and manufacturing complexity
Size and shape	Ultra-small stomatocytes, spherical and cylindrical nanomotors	Affect tissue penetration, biodistribution, cellular uptake, and propulsion efficiency	Enhanced transport across biological barriers and improved target accumulation	Trade-off between cargo capacity and tissue penetration
Enzyme immobilization	Encapsulation, covalent immobilization, non-covalent adsorption, surface functionalization	Regulates enzyme stability, catalytic activity, and substrate accessibility	Improved enzyme protection and prolonged activity	Possible reduction in catalytic activity after immobilization
Surface functionalization	Magnetic nanoparticles, carbon-based materials, plant-derived biopolymers (e.g., chitosan, lignin)	Enhances targeting capability and interaction with biological environments	Improved selectivity, stability, and biocompatibility	Increased fabrication complexity
Motion control and swarms	Chemical gradients, magnetic fields, light-responsive systems, collective swarming behavior	Enables navigation, directional control, and coordinated cargo transport	Precise guidance and enhanced transport efficiency	Limited control under complex physiological conditions

**Table 3:** Representative preclinical studies of enzymatic nanomotors for cancer therapy.

Type of Enzymatic Nanomotor	Experimental Model	Therapeutic Mechanism	Main Biological Outcome	Translational Challenge	Ref.
Urease-powered mesoporous silica nanobots loaded with doxorubicin	HeLa cell	Active urease-driven propulsion enhances intracellular drug transport	Active urease-driven propulsion enhances intracellular drug transport	Limited control of propulsion in vivo	[132]
Urease-powered nanobots for radionuclide delivery	Orthotopic mouse model of bladder cancer	Active propulsion improves intravesical distribution and retention of therapeutic agents	Enhanced radionuclide delivery and therapeutic efficacy	Long-term biodistribution and safety require validation	[133]
GOx/CAT- powered cell membrane-camouflaged nanomotors	4T1 breast cancer cells, multicellular tumor spheroids, 4T1 tumor-bearing mice	Chemotaxis toward glucose and pH gradients combined with homologous tumor targeting	Enhanced tumor accumulation, deeper tumor penetration, and improved antitumor efficacy	Manufacturing complexity and scalability	[134]
GOx/CAT-powered prodrug-skeletal ZIF nanomotors	4T1 breast cancer cells, multicellular tumor spheroids, and 4T1 tumor-bearing mice	Autonomous propulsion combined with synergistic chemo/starvation/photodynamic therapy	Enhanced tumor accumulation and superior antitumor efficacy	Clinical translation and reproducibility	[49]
Catalase-powered ultrasmall stomatocyte nanomotors	HeLa cells and endothelial vasculature model	Catalase-driven self-propulsion enhances vascular penetration and cellular uptake	Enhanced penetration across vasculature models and increased cellular uptake	Requirement for exogenous H_2_O_2_ fuel and in vivo validation	[135]

Abbreviations: GOx—glucose oxidase; CAT—catalase; ZIF—zeolitic imidazolate framework.

**Table 4:** Potential neurological applications of enzymatic nanomotors and phytochemi-cal-based nanosystems.

Type of Nanosystem	Experimental Model	Therapeutic Mechanism	Main Biological Outcome	Translational Challenge	Ref.
Nitric oxide-driven chemotactic nanomotors	GL261 and U87 glioblastoma cells, in vitro BBB model, and orthotopic glioblastoma mouse model	ROS/iNOS-responsive chemotaxis enhances BBB penetration, tumor targeting, and immunotherapy	Enhanced BBB transport, glioblastoma accumulation, T-cell infiltration, and antitumor efficacy	Long-term biosafety, manufacturing scalability, and clinical translation	[136]
Curcumin-loaded PLGA nanoparticles	Neural stem cells, A*β*-induced AD rat model	Activation of canonical Wnt/*β*-catenin signaling and enhancement of neurogenesis	Induced neurogenesis and reversed learning and memory deficits	Limited clinical validation and large scale manufacturing considerations	[137]
Quercetin-loaded *β*-cyclodextrin-dodecylcarbonate nanoparticles	SH-SY5Y neuroblastoma cells exposed to AD-related oxysterols	Enhanced cellular delivery of and bioavailability of quercetin leading to inhibition of TLR4/COX-2 inflammatory signaling	Reduced neuroinflammatory mediator expression and enhanced anti-inflammatory efficacy compared with free quercetin	Limited clinical translation	[138]
Resveratrol-loaded OX26-functionalized solid lipid nanoparticles	Human in vitro BBB model and A*β*(1–42) aggregation assays	Transferrin receptor-mediated BBB transport and enhanced brain delivery of resveratrol	Improved BBB transcytosis and inhibition of amyloid-*β* aggregation	Limited in vivo and clinical validation	[139]
EGCG nanoparticles	Aluminum chloride-induced rat model of AD	Enhanced bioavailability of EGCG and inhibition of A*β* aggregation, tau pathology, and oxidative stress	Attenuated neurobehavioral deficits and reduced A*β* and tau pathology	Limited clinical validation and large-scale manufacturing	[140]

Abbreviations: BBB—blood–brain barrier; ROS—reactive oxygen species; iNOS—inducible nitric oxide synthase; PLGA—poly (lactic-co-glycolic acid; A*β*—amyloid-beta; AD—Alzheimer’s disease; EGCGepigallocatechin gallate.

**Table 5:** Potential applications of enzymatic nanomotors and phytochemical-based nanosystems in inflammatory diseases.

Strategy	Potential Inflammatory Disease Application	Proposed Therapeutic Benefit	Main Limitation	Ref.
Catalase-powered nanomotors	ROS-associated inflammatory disorders	Reduction in oxidative stress through hydrogen peroxide decomposition	Limited long-term biosafety data	[2]
ROS-responsive enzymatic nanosystems	Chronic inflammatory microenvironments	Controlled therapeutic release in response to elevated ROS levels	Potential imbalance in redox homeostasis	[141]
Curcumin-loaded nanocarriers	Inflammatory bowel disease (ulcerative colitis)	Suppression of NF-κB signaling, reduction in pro-inflammatory cytokines, and modulation of gut microbiota	Limited clinical validation and formulation-dependent bioavailability	[142,143,144]
Enzyme-assisted active nanocarriers	Localized inflammatory lesions	Enhanced tissue penetration and local therapeutic retention	Motion control under physiological conditions	[2]
Biohybrid membrane-coated nanocarriers	Precision anti-inflammatory therapy	Improved circulation time and reduced immune clearance	Manufacturing reproducibility and regulatory complexity	[135]

Abbreviations: ROS—reactive oxygen species.

**Table 6:** Potential applications of enzymatic nanomotors and phytochemical-based nanosystems in regenerative medicine.

Strategy	Potential Regenerative Medicine Application	Proposed Therapeutic Benefit	Main Limitation	Ref.
Enzyme-powered nanomotors	Chronic wound healing	Enhanced tissue penetration and localized therapeutic delivery	Limited in vivo validation	[2,26]
ROS-responsive enzymatic nanosystems	Oxidative stress-associated tissue injury	Controlled release in ROS-rich microenvironments and reduction in oxidative stress	Potential imbalance in redox homeostasis	[144]
Polyphenol-loaded nanocarriers	Wound healing and tissue repair	Enhanced wound healing through antioxidant and anti-inflammatory activity, improved collagen deposition, and accelerated tissue regeneration	Limited clinical validation and poor phytochemical stability/bioavailability	[141]
Biohybrid membrane-coated nanocarriers	Precision regenerative therapy	Improved circulation time and enhanced biocompatibility	Manufacturing reproducibility and regulatory complexity	[135]
Enzyme-assisted active nanocarriers	Localized tissue regeneration	Improved retention within damaged tissues and enhanced therapeutic penetration	Motion control under physiological conditions	[26]

## Data Availability

The data presented in this study are available on request from the corresponding author.
